# Manganese(II) chloride complexes with pyridine *N*-oxide (PNO) derivatives and their solid-state structures

**DOI:** 10.1107/S2056989017012038

**Published:** 2017-09-12

**Authors:** Linda Kang, Genevieve Lynch, Will Lynch, Clifford Padgett

**Affiliations:** a Department of Chemistry and Physics, Armstrong State University, Savannah, Georgia 31419, USA; b St Vincent’s Academy, Savannah, Georgia 31401, USA

**Keywords:** crystal structure, manganese, pyridine *N*-oxide ligand

## Abstract

Three manganese(II) *N*-oxide complexes have been synthesized from the reaction of manganese(II) chloride with either pyridine *N*-oxide (PNO), 2-methyl­pyridine *N*-oxide (2MePNO) or 3-methyl­pyridine *N*-oxide (3MePNO). The compounds were synthesized from methano­lic solutions of MnCl_2_·4H_2_O and the respective *N*-oxide, and subsequently characterized structurally by single-crystal X-ray diffraction. The compounds are *catena*-poly[[aqua­chlorido­manganese(II)]-di-μ-chlorido-[aqua­chlorido­manganese(II)]-bis­(μ-pyridine *N*-oxide)], [MnCl_2_(C_5_H_5_NO)(H_2_O)]_*n*_ or [MnCl_2_(PNO)(H_2_O)]_*n*_ (**I**), *catena*-poly[[aqua­chlorido­man­gan­ese(II)]-di-μ-chlorido-[aqua­chlorido­manganese(II)]-bis­(μ-2-methyl­pyridine *N*-oxide)], [MnCl_2_(C_6_H_7_NO)(H_2_O)]_*n*_ or [MnCl_2_(2MePNO)(H_2_O)]_*n*_ (**II**), and bis­(μ-3-methyl­pyridine *N*-oxide)bis­[di­aqua­dichlorido­manganese(II)], [Mn_2_Cl_4_(C_6_H_7_NO)_2_(H_2_O)_4_] or [MnCl_2_(3MePNO)(H_2_O)_2_]_2_ (**III**). The Mn^II^ atoms are found in pseudo-octa­hedral environments for each of the three complexes. Compound **I** forms a coordination polymer with alternating pairs of bridging *N*-oxide and chloride ligands. The coordination environment is defined by two PNO bridging O atoms, two chloride bridging atoms, a terminal chloride, and a terminal water. Compound **II** also forms a coordination polymer with a similar metal cation; however, the coordination polymer is bridged between Mn^II^ atoms by both a single chloride and 2MePNO. The distorted octahedrally coordinated metal cation is defined by two bridging 2MePNO *trans* to each other, two chlorides, also *trans* to one another in the equatorial (polymeric) plane, and a terminal chloride and terminal water. Finally, complex **III** forms a dimer with two bridging 3MePNOs, two terminal chlorides and two terminal waters forming the six-coordinate metal environment. All three compounds exhibit hydrogen bonding between the coordinating water(s) and terminal chlorides.

## Abstract 2

The synthesis and structures of three manganese(II) pyridine *N*-oxide complexes are presented.

## Chemical context   

The utility of aromatic *N*-oxides to facilitate organic oxo-transfer reactions has been well documented over the years (see, for example, Eppenson, 2003 ▸). Many of these reactions are actually catalyzed by transition metal inter­actions with the *N*-oxide ligands (see, for example, Moustafa *et al.*, 2014 ▸). Furthermore, *N*-oxide metal inter­actions have recently attracted much inter­est in a variety of other areas, including metal organic frameworks (MOFs) (Hu *et al.*, 2014 ▸). These MOFs synthesized using *N*-oxide derivatives take advantage of the multiple binding modes of the *sp* ^3^ O atom and the ease of modification of the organic backbone of the *N*-oxide. The utility of the MOFs has been examined in areas such as catalysis (Liu *et al.*, 2014 ▸) and sensors (Hu *et al.*, 2014 ▸). The constructs extend to the supra­molecular study of coordination polymers that have been found in this type of complex because of their incredible versatility as ligands (Sarma & Baruah, 2011 ▸).

In this context, we report the synthesis and solid-state structures of three pyridine *N*-oxide manganese(II) com­plexes. Notably, we used the ligands pyridine *N*-oxide, 2-methyl­pyridine *N*-oxide, and 3-methyl­pyridine *N*-oxide to study the impact of substitution of the pyridine on the two- and three-dimensional solid-state structures. The pyridine *N*-oxide (PNO) and 2-methyl­pyridine *N*-oxide (2MePNO) complexes form coordination polymers with subtle differences. The 3-methyl­pyridine *N*-oxide (3MePNO), however, forms a dimeric complex.
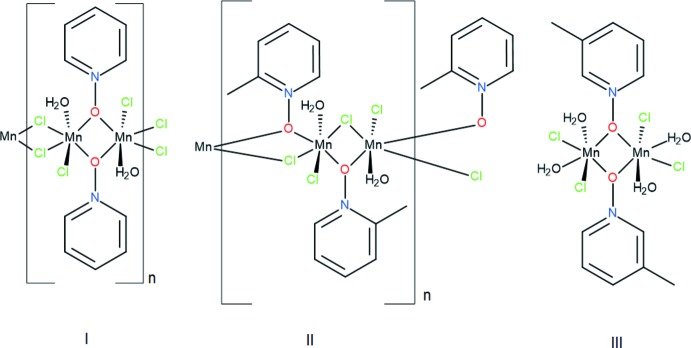


## Structural commentary   

**Figure 1:**
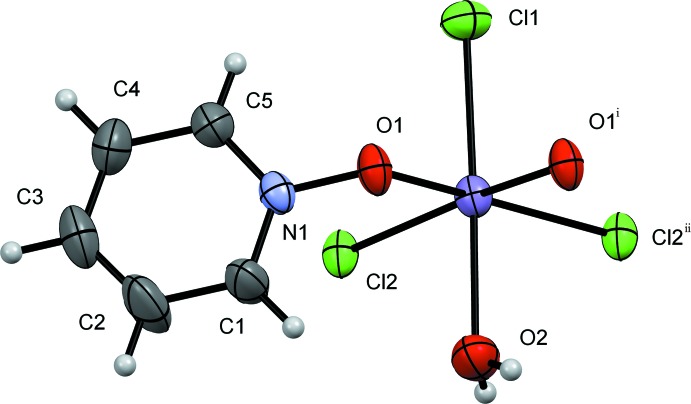
A view of compound **I**, showing the atom labeling. Displacement ellipsoids are drawn at the 50% probability level. [Symmetry codes: (i) −*x* + 1, −*y* + 1, −*z* + 1; (ii) −*x*, -*y+*1, −*z* + 1]

**Figure 2:**
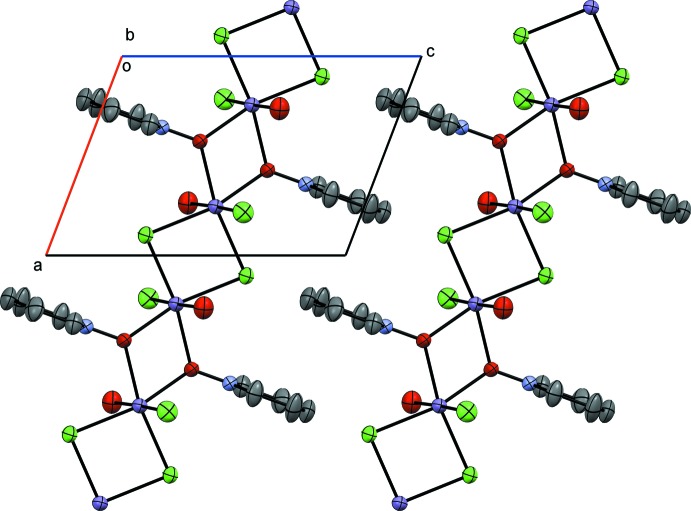
Crystal packing diagram of compound **I**, viewed along the *b* axis. H atoms have been omitted for clarity.

Complex **I** exhibits the repeating motif of [MnCl_2_(PNO)(H_2_O)]_*n*_ and crystallizes in the triclinic space group *P* 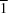, containing two formula units per unit cell (Fig. 1 ▸). The coordination sphere around each Mn^II^ atom is a distorted octa­hedron, with the equatorial atoms being two bridging chlorides alternating with two bridging pyridine *N*-oxide (PNO) mol­ecules (Fig. 2 ▸). In the equatorial plane, the bridging chlorides and the bridging pyridine *N*-oxides are *cis* to one another. The axial positions are a terminal chloride and a water molecule. The Mn1—O1 bond length is 2.177 (3) Å, whereas the Mn1—O1^vii^ bond length is slightly longer at 2.182 (3) Å for the bridging PNO [symmetry code (vii) −*x* + 1, −*y* + 1, −*z* + 1]. The bridging chlorides are found to have Mn—Cl2 distances of 2.5240 (19) and 2.532 (19) Å, respectively. Axially, the water is located 2.250 (3) Å from the Mn^II^ cation and the terminal chloride is at 2.479 (2) Å. The bond angles around the equator are severely compressed at the two bridging *N*-oxides, with the O1—Mn1—O1^i^ angle observed at 72.03 (10)°. The remaining three angles are found to all be similar at 95.58 (7) (Cl2—Mn1—Cl2^i^), 96.80 (8) (O1—Mn1—Cl2), and 94.69 (9)° (O1^vii^—Mn1—Cl2^vii^). Axially, the bond angle from the water through manganese(II) and the terminal chloride (O2—Mn1—Cl1) is nearly linear at 177.36 (8)°.

**Figure 3:**
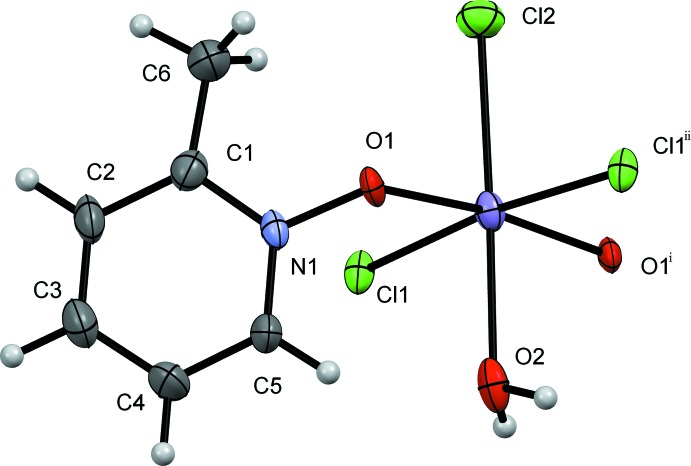
A view of compound **II**, showing the atom labeling. Displacement ellipsoids are drawn at the 50% probability level. [Symmetry codes: (i) −*x* − 1, *y* + 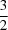, −*z* + 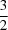; (ii) −*x*, *y* + 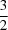, −*z* + 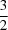.]

Complex **II**, [MnCl_2_(2MePNO)(H_2_O)]_*n*_, posseses a metal environment similar to complex **I** and crystallizes in the ortho­rhom­bic space group *P*2_1_2_1_2_1_. The major difference in structure **II** is in the bridging network, where the chlorides and *N*-oxides are *trans* to one another rather than *cis* as in **I** (Figs. 3 ▸ and 4 ▸). The pseudo-octa­hedral environment includes an Mn1—Cl1 bond length of 2.516 (4) Å and an Mn1—O1 (*N*-oxide) bond length of 2.170 (6) Å, with a Cl1—Mn1—O1 bond angle of 84.37 (19)°. The bond angle across the Cl atoms, Cl1—Mn1—Cl1^viii^, is 174.02 (5)° and across the O atoms of 2MePNO, O1—Mn1—O1^ix^, is 173.12 (6)°; a slight compression is observed across the bridges [symmetry codes: (viii) −*x* − 1, *y* + 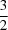, −*z* + 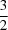; (ix) −*x*, *y* + 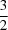, −*z* + 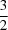]. The axial (non-bridging) Mn1—Cl2 bond length is 2.503 (4) Å, while the axial water is found at a distance of 2.268 (6) Å from the metal center.

The dimeric complex **III**, [MnCl_2_(3MePNO)(OH_2_)_2_]_2_, crystallizes in the triclinic *P* 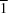 space group, with the inversion center sitting in the center of the dimer (Fig. 5 ▸). The 3-methyl derivative does not form a coordination polymer but discrete dimeric mol­ecules. The structure possesses two bridging 3MePNO ligands, four terminal chlorides, and four terminal waters. Two waters and two chlorides are in the equatorial plane coincident with the *N*-oxide bridge, and the other equivalents are axial in the pseudo-octa­hedral geometry around the Mn^II^ atoms. The Mn1—Cl1 and Mn1—Cl2 bond lengths are 2.4601 (5) and 2.4903 (19) Å, respectively, with a Cl1—Mn1—Cl2 bond angle of 98.32 (4)°. The bridging *N*-oxide is at a distance of 2.1791 (18) Å from Mn1—O1, with an O1—Mn1—O1^vii^ bond angle of 71.86 (7)° [symmetry code: (vii) −*x* + 1, −*y* + 1, −*z* + 1]. The Mn1—O2(water) and Mn1—O3(water) bond lengths are 2.245 (2) and 2.1696 (17) Å, respectively, with an O2—Mn1—O3 bond angle of 85.83 (7)°.

The formation of the polymeric structure in **I** and **II** *versus* the dimer in **III** is likely due to the steric influence of the methyl group in the 3-position in 3MePNO and the core constituents. One can define the Mn_2_ ‘*N*-oxide diamond core’ in each of the structures as follows: **I** is alternating Mn_2_Cl_2_ and Mn_2_O_2_ (oxygen bridges *via* PNO) cores, **II** is Mn_2_ClO (oxygen bridge *via* 2MePNO) and **III** Mn_2_O_2_ (oxygen bridges *via* 3MePNO). In **I**, the unsubstituted pyridine *N*-oxide group does not generate as much steric strain, allowing for polymer formation. In **II**, the core is formed to permit alternating up and down pyridine *N*-oxides with the 2-methyl substituents also facing in opposite directions. This limits the steric inter­actions and the *N*-oxide slightly tilts out of the polymeric core line to allow the methyl group to effect less steric inter­actions. In **III**, the methyl group appears to inhibit polymer formation due to the position of this bulky substituent. Subsequently, when the polymer is not formed, an extra water mol­ecule is required to fill the sixth coordination site on the metal cation occupied by a bridging atom in **I** and **II**.

## Supra­molecular features   

The packing of **I** forms a coordination polymer of alternating bis-bridges of two chlorides and two pyridine *N*-oxides in the *a*-axis direction (Fig. 2 ▸). The aromatic rings stack at 6.860 (7) Å, outside of π-stacking distance due to the alternating chloride and pyridine *N*-oxide bridges. The single water mol­ecule is locked into weak hydrogen-bonding inter­actions in two different modes. One hydrogen-bond inter­action (H2*A*) is located down the bridge to the terminal chloride (Cl1), on the adjacent Mn^II^ atom, and the O2—H2*A*⋯Cl1^i^ distance is 2.53 (2) Å. The other hydrogen-bond inter­action (H2*B*) is across to the next polymeric chain with Cl1; the O2—H2*B*⋯Cl1^ii^ distance is 2.52 (3) Å (see Table 1 ▸ for hydrogen-bond details and symmetry codes).

**Figure 4:**
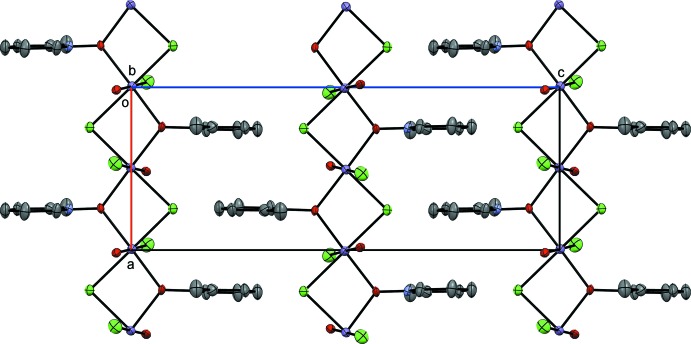
Crystal packing diagram of compound **II**, viewed along the *b* axis. H atoms have been omitted for clarity.

Complex **II** packs as a coordination polymer in the *a* direction similar to **I** (Fig. 4 ▸). However, as **I** has alternating pyridine *N*-oxide and chloride bridges (placing these ligands *cis* to one another), **II** has a single 2-methyl­pyridine *N*-oxide and a single chloride in each bridge. Similar to **I**, the hydrogen-bonding inter­actions are to a terminal chloride (Cl2) on the adjacent Mn^II^ atom. There are two observed inter­actions, *viz.* O2—H2*A*⋯Cl2^iii^ with a distance of 2.49 Å and O2—H2*B*⋯Cl2^iv^ with a distance of 2.26 Å (see Table 2 ▸ for hydrogen-bond details and symmetry codes). The H2*A*⋯ Cl2 inter­action is in the coordination polymer and the H2*B*⋯Cl2 inter­action is across the polymeric chains. Similar to **I**, the aromatic rings stack too far apart to be inter­acting in the *a* direction, at a distance of 6.862 (11) Å.

**Figure 5:**
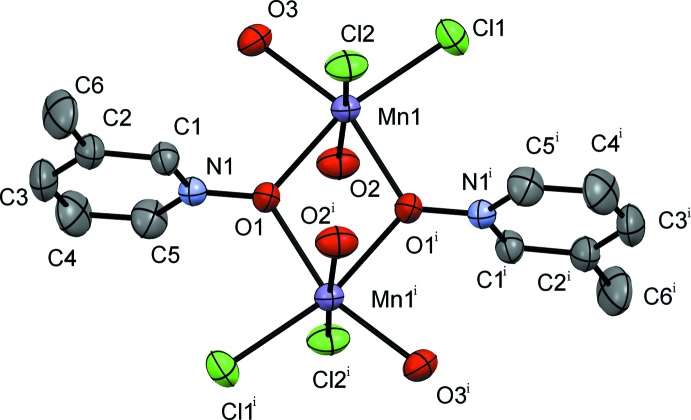
A view of the mol­ecular structure of compound **III**, showing the atom labeling. Displacement ellipsoids are drawn at the 50% probability level. H atoms have been omitted for clarity. [Symmetry code: (i) −*x* − 1, −*y* + 1, −*z* + 1.]

As noted above, compound **III** does not form a coordination polymer but is observed in the solid state as a dimer with two water mol­ecules for each Mn^II^ atom (*versus* one aqua equivalent in **I** and **II**) (Fig. 5 ▸). The aromatic inter-centroid distance is longer than in the other two mol­ecules, at 7.902 (7) Å. In compound **III**, a single water mol­ecule hydrogen bonds from the equatorial plane of one dimer to an axial chloride on another dimer. Conversely, the axial water hydrogen bonds to an equatorial chloride on a different dimer. These inter­actions are found to be O2—H2*B*⋯Cl1^v^ [distance 2.38 (2) Å] and O3—H3*A*⋯Cl2^vi^ [distance 2.28 (2) Å] (see Table 3 ▸ for hydrogen-bond details and symmetry codes).

## Database survey   

A search in the Cambridge Structural Database (CSD; Groom *et al.*, 2016 ▸) for aromatic *N*-oxides bound to manganese returned 87 entries. Similar *N*-oxides with simple counter-ions in the list include 4,4′-di­pyridine *N*,*N*′-dioxide [FIVHAU (Ma *et al.*, 2005 ▸) and XOHQUH (Jiu *et al.*, 2008 ▸)], 1,2-bis­(4-pyrid­yl)ethane *N*,*N*′-dioxide (TOJDAY and TOJDIG; Sun *et al.*, 2008 ▸), and 1,3-bis­(4-pyrid­yl)propane *N*,*N*′-dioxide (Zhang *et al.*, 2003 ▸). Similarly, two derivatives of 3,5-di­methyl­pyridine *N*-oxide are found in the CSD (GIWQAF and GIWQEJ; Shi *et al.*, 2007 ▸)

## Synthesis and crystallization   

The title compounds were all synthesized in a similar manner. 0.200 g of MnCl_2_·4H_2_O (1.01 mmol) was dissolved in a minimal amount of methanol, approximately 10 ml. Two stoichiometric equivalents of the appropriate *N*-oxide were also dissolved in approximately 20 ml of methanol (PNO: 0.191 g, 2.02 mmol; 2MePNO: 0.220 g, 2.02 mmol; 3MePNO: 0.220 g, 2.02 mmol). The solutions were stirred for approximately 10 min; during each reaction, a brown solution was observed upon mixing. The reaction solution was then allowed to sit and brown crystals were grown by slow evaporation in the near qu­anti­tative yields reported below based on the manganese(II) chloride starting material. The FT–IR spectra of the complexes all exhibit broad absorbances in the 3400–3000 cm^−1^ region due to the ν(O—H) of the coordinating water mol­ecules, as well as the characteristic ν(N—O) of the *N*-oxide pyridyl moiety in the 1250–1150 cm^−1^ region noted previously (Mautner *et al.*, 2017 ▸).

Compound **I**, Mn(PNO)Cl_2_·H_2_O, yield 0.215 g (90.3%). Selected IR bands (ATR, FT–IR, KBr composite, cm^−1^): 3364 (*m*, *br*), 3235 (*m*, *br*), 3068 (*m*, *br*), 1660 (*w*), 1471 (*w*), 1214 (*m*), 1205 (*m*), 1023 (*s*), 831 (*s*), 780 (*s*), 674 (*s*), 556 (*s*). Elemental analysis for MnCl_2_C_5_H_7_NO_2_, calculated (%): C 25.13, H 2.95, N 5.86; found (%): C 25.22, H 2.96, N 5.87.

Compound **II**, Mn(2MePNO)Cl_2_·H_2_O, yield 0.227 g (87.9%). Selected IR bands (ATR, FT–IR, KBr composite, cm^−1^): 3410 (*m*, *br*), 3247(*m*, *br*), 3073(*m*, *br*), 1716 (*w*), 1619 (*w*), 1578 (*w*), 1421 (*m*), 1264 (*m*), 1154 (*m*), 1029 (*s*), 831 (*s*), 799 (*s*), 684 (*s*), 584 (*s*). Elemental analysis for MnCl_2_C_6_H_9_NO_2_, calculated (%): C 28.48, H 3.59, N 5.53; found (%): C 28.75, H 3.53, N 5.28.

Compound **III**, Mn_2_(3MePNO)_2_Cl_4_·4H_2_O, yield 0.231 g (89.5%). Selected IR bands (ATR, FT–IR, KBr composite, cm^−1^): 3374 (*m*, *br*), 3251 (*m*, *br*), 3094 (*m*, *br*), 1663 (*w*), 1614(*w)*, 1492 (*m*), 1261 (*m*), 1164 (*m*), 1019 (*s*), 946 (*s*), 750 (*s*), 672 (*s*). Elemental analysis for Mn_2_Cl_4_C_12_H_22_N_2_O_6_, calculated (%): C 26.59, H 4.09, N 5.16; found (%): C 26.53, H 4.04, N 5.21.

## Refinement   

Crystal data, data collection and structure refinement details are summarized in Table 4 ▸. All carbon-bound H atoms were positioned geometrically and refined as riding, with C—H = 0.95 or 0.98 Å and *U*_iso_(H) = 1.2*U*_eq_(C) or *U*_iso_(H) = 1.5*U*_eq_(C) for C(H) and CH_3_ groups, respectively. In order to ensure chemically meaningful O—H distances for the bound water mol­ecules in compound **I**, the H2*A*—O2 and H2*B*—O2 distances were restrained to a target value of 0.84 (2) Å (using a DFIX command in *SHELXL2017*; Sheldrick, 2015*b* ▸). In compound **II**, water H atoms were refined as riding, with the O—H distance constrained to 0.892 Å and *U*_iso_(H) = 1.5*U*_eq_(O) using an AFIX 7 command, and in compound **III**, H2*A*—O2, H2*B*—O2, H3*A*—O3, and H3*B*—O3 were restrained using DFIX as for compound **I**. A rotating-group model was applied for the methyl groups. Structure refinement of **II** exhibits inversion twinning. Several crystals were tried and the centrosymmetric space group *Pnma* was tested. In all cases, there was a significant reduction in the *R* value for the inversion twinning *P*2_1_2_1_2_1_ solution.

## Supplementary Material

Crystal structure: contains datablock(s) I, II, III. DOI: 10.1107/S2056989017012038/zl2709sup1.cif

Structure factors: contains datablock(s) I. DOI: 10.1107/S2056989017012038/zl2709Isup2.hkl

Structure factors: contains datablock(s) II. DOI: 10.1107/S2056989017012038/zl2709IIsup3.hkl

Structure factors: contains datablock(s) III. DOI: 10.1107/S2056989017012038/zl2709IIIsup4.hkl

CCDC references: 1570001, 1570000, 1569999

Additional supporting information:  crystallographic information; 3D view; checkCIF report

## Figures and Tables

**Table 1:** Hydrogen-bond geometry (Å, °) for **I** [Chem scheme1]

*D*—H⋯*A*	*D*—H	H⋯*A*	*D*⋯*A*	*D*—H⋯*A*
O2—H2*A*⋯Cl1^i^	0.83 (2)	2.53 (2)	3.348 (4)	168 (4)
O2—H2*B*⋯Cl1^ii^	0.82 (2)	2.52 (3)	3.232 (4)	147 (4)

Symmetry codes: (i) 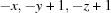; (ii) 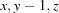.

**Table 2:** Hydrogen-bond geometry (Å, °) for **II** [Chem scheme1]

*D*—H⋯*A*	*D*—H	H⋯*A*	*D*⋯*A*	*D*—H⋯*A*
O2—H2*A*⋯Cl2^iii^	0.90	2.49	3.205 (7)	137
O2—H2*B*⋯Cl2^iv^	0.89	2.26	3.145 (7)	169

Symmetry codes: (iii) 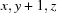; (iv) 
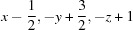
.

**Table 3:** Hydrogen-bond geometry (Å, °) for **III** [Chem scheme1]

*D*—H⋯*A*	*D*—H	H⋯*A*	*D*⋯*A*	*D*—H⋯*A*
O2—H2*B*⋯Cl1^v^	0.80 (2)	2.38 (2)	3.147 (3)	161 (2)
O3—H3*A*⋯Cl2^vi^	0.86 (2)	2.28 (2)	3.120 (2)	167 (2)

Symmetry codes: (v) 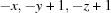; (vi) 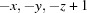.

**Table 4:** Experimental details

	**I**	**II**	**III**
Crystal data			
Chemical formula	[MnCl_2_(C_5_H_5_NO)(H_2_O)]	[MnCl_2_(C_6_H_7_NO)(H_2_O)]	[Mn_2_Cl_4_(C_6_H_7_NO)_2_(H_2_O)_4_]
*M*_r_	238.96	252.98	541.99
Crystal system, space group	Triclinic, *P* 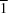	Orthorhombic, *P*2_1_2_1_2_1_	Triclinic, *P* 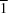
Temperature (K)	173	173	173
*a*, *b*, *c* (Å)	6.897 (2), 7.050 (1), 9.853 (3)	6.862 (2), 7.491 (2), 18.047 (5)	7.902 (7), 8.026 (7), 9.893 (8)
α, β, γ (°)	101.042 (7), 109.559 (10), 94.196 (6)	90, 90, 90	98.033 (1), 99.272 (7), 113.634 (11)
*V* (Å^3^)	438.2 (2)	927.7 (4)	552.6 (8)
*Z*	2	4	1
Radiation type	Mo *K*α	Mo *K*α	Mo *K*α
μ (mm^−1^)	2.06	1.96	1.65
Crystal size (mm)	0.29 × 0.18 × 0.13	0.2 × 0.2 × 0.1	0.85 × 0.50 × 0.28

Data collection			
Diffractometer	Rigaku XtalLab mini CCD	Rigaku XtalLab mini CCD	Rigaku XtalLab mini CCD
Absorption correction	Multi-scan (*REQAB*; Rigaku, 1998 ▸)	Multi-scan (*REQAB*; Rigaku, 1998 ▸)	Multi-scan (*REQAB*; Rigaku, 1998 ▸)
*T*_min_, *T*_max_	0.613, 0.765	0.563, 0.737	0.482, 0.630
No. of measured, independent and observed [*I* > 2σ(*I*)] reflections	4655, 2004, 1770	8438, 2109, 1800	5837, 2553, 2375
*R*_int_	0.040	0.051	0.072
(sin θ/λ)_max_ (Å^−1^)	0.651	0.649	0.652

Refinement			
*R*[*F* ^2^ > 2σ(*F* ^2^)], *wR*(*F* ^2^), *S*	0.031, 0.080, 1.14	0.051, 0.100, 1.12	0.031, 0.087, 1.07
No. of reflections	2004	2109	2553
No. of parameters	108	112	135
No. of restraints	2	0	4
H-atom treatment	H atoms treated by a mixture of independent and constrained refinement	H-atom parameters constrained	H atoms treated by a mixture of independent and constrained refinement
Δρ_max_, Δρ_min_ (e Å^−3^)	0.40, −0.44	0.95, −0.73	0.56, −0.41
Absolute structure	–	Refined as an inversion twin	–
Absolute structure parameter	–	0.44 (8)	–

Computer programs: *CrystalClearSM Expert* (Rigaku, 2011 ▸), *SHELXT* (Sheldrick, 2015*a* ▸), *SHELXL2017* (Sheldrick, 2015*b* ▸) and *OLEX2* (Dolomanov *et al.*, 2009 ▸).

## supplementary crystallographic information

### catena-poly[[aquachloridomanganese(II)]-di-µ-chlorido-[aquachloridomanganese(II)]-bis(µ-pyridine N-oxide)] (I) Crystal data

**Table:**

[MnCl_2_(C_5_H_5_NO)(H_2_O)]	*Z* = 2
*M**_r_* = 238.96	*F*(000) = 238
Triclinic, *P*1	*D*_x_ = 1.811 Mg m^−^^3^
*a* = 6.897 (2) Å	Mo *K*α radiation, λ = 0.71073 Å
*b* = 7.050 (1) Å	Cell parameters from 1189 reflections
*c* = 9.853 (3) Å	θ = 2.3–27.5°
α = 101.042 (7)°	µ = 2.06 mm^−^^1^
β = 109.559 (10)°	*T* = 173 K
γ = 94.196 (6)°	Prism, clear brown
*V* = 438.2 (2) Å^3^	0.29 × 0.18 × 0.13 mm

### catena-poly[[aquachloridomanganese(II)]-di-µ-chlorido-[aquachloridomanganese(II)]-bis(µ-pyridine N-oxide)] (I) Data collection

**Table:**

Rigaku XtalLab mini CCD diffractometer	1770 reflections with *I* > 2σ(*I*)
ω scans	*R*_int_ = 0.040
Absorption correction: multi-scan (REQAB; Rigaku, 1998)	θ_max_ = 27.6°, θ_min_ = 2.3°
*T*_min_ = 0.613, *T*_max_ = 0.765	*h* = −8→8
4655 measured reflections	*k* = −9→9
2004 independent reflections	*l* = −12→12

### catena-poly[[aquachloridomanganese(II)]-di-µ-chlorido-[aquachloridomanganese(II)]-bis(µ-pyridine N-oxide)] (I) Refinement

**Table:**

Refinement on *F*^2^	Primary atom site location: dual
Least-squares matrix: full	Hydrogen site location: mixed
*R*[*F*^2^ > 2σ(*F*^2^)] = 0.031	H atoms treated by a mixture of independent and constrained refinement
*wR*(*F*^2^) = 0.080	*w* = 1/[σ^2^(*F*_o_^2^) + 0.444*P*] where *P* = (*F*_o_^2^ + 2*F*_c_^2^)/3
*S* = 1.14	(Δ/σ)_max_ = 0.001
2004 reflections	Δρ_max_ = 0.40 e Å^−^^3^
108 parameters	Δρ_min_ = −0.44 e Å^−^^3^
2 restraints	

### catena-poly[[aquachloridomanganese(II)]-di-µ-chlorido-[aquachloridomanganese(II)]-bis(µ-pyridine N-oxide)] (I) Special details

**Table:**

Geometry. All esds (except the esd in the dihedral angle between two l.s. planes) are estimated using the full covariance matrix. The cell esds are taken into account individually in the estimation of esds in distances, angles and torsion angles; correlations between esds in cell parameters are only used when they are defined by crystal symmetry. An approximate (isotropic) treatment of cell esds is used for estimating esds involving l.s. planes.

### catena-poly[[aquachloridomanganese(II)]-di-µ-chlorido-[aquachloridomanganese(II)]-bis(µ-pyridine N-oxide)] (I) Fractional atomic coordinates and isotropic or equivalent isotropic displacement parameters (Å^2^)

**Table:**

	*x*	*y*	*z*	*U*_iso_*/*U*_eq_	
Mn1	0.24478 (6)	0.51944 (6)	0.49588 (4)	0.02748 (12)	
Cl1	0.21608 (12)	0.81797 (10)	0.39668 (9)	0.04150 (19)	
O1	0.5727 (3)	0.6094 (3)	0.63106 (19)	0.0327 (4)	
N1	0.6425 (3)	0.6894 (3)	0.7767 (2)	0.0283 (5)	
C1	0.6940 (6)	0.5762 (5)	0.8731 (3)	0.0493 (8)	
H1	0.681940	0.441595	0.840110	0.059*	
Cl2	0.10246 (10)	0.65268 (10)	0.69397 (7)	0.03317 (16)	
O2	0.2634 (4)	0.2541 (3)	0.5927 (3)	0.0447 (5)	
H2A	0.146 (4)	0.218 (6)	0.594 (5)	0.081 (16)*	
H2B	0.305 (7)	0.162 (5)	0.551 (5)	0.087 (16)*	
C2	0.7655 (7)	0.6608 (6)	1.0222 (4)	0.0655 (11)	
H2	0.802254	0.582800	1.090313	0.079*	
C3	0.7826 (6)	0.8564 (6)	1.0704 (3)	0.0543 (9)	
H3	0.829678	0.913307	1.171227	0.065*	
C4	0.7299 (6)	0.9692 (5)	0.9693 (4)	0.0532 (9)	
H4	0.742760	1.104037	1.001068	0.064*	
C5	0.6577 (5)	0.8836 (5)	0.8203 (3)	0.0421 (7)	
H5	0.619974	0.959566	0.750808	0.051*	

### catena-poly[[aquachloridomanganese(II)]-di-µ-chlorido-[aquachloridomanganese(II)]-bis(µ-pyridine N-oxide)] (I) Atomic displacement parameters (Å^2^)

**Table:**

	*U*^11^	*U*^22^	*U*^33^	*U*^12^	*U*^13^	*U*^23^
Mn1	0.0228 (2)	0.0328 (2)	0.0259 (2)	0.00479 (16)	0.00858 (16)	0.00467 (16)
Cl1	0.0478 (4)	0.0329 (4)	0.0481 (4)	0.0073 (3)	0.0188 (4)	0.0156 (3)
O1	0.0230 (9)	0.0460 (12)	0.0238 (9)	0.0052 (8)	0.0062 (8)	−0.0006 (8)
N1	0.0232 (11)	0.0371 (12)	0.0233 (10)	0.0033 (9)	0.0078 (9)	0.0052 (9)
C1	0.076 (2)	0.0386 (17)	0.0325 (15)	0.0152 (16)	0.0152 (16)	0.0124 (13)
Cl2	0.0307 (3)	0.0377 (4)	0.0267 (3)	0.0016 (3)	0.0102 (3)	−0.0016 (3)
O2	0.0553 (16)	0.0394 (13)	0.0420 (13)	0.0117 (11)	0.0186 (12)	0.0115 (10)
C2	0.094 (3)	0.073 (3)	0.0290 (16)	0.031 (2)	0.0135 (19)	0.0204 (17)
C3	0.057 (2)	0.071 (3)	0.0249 (15)	0.0074 (18)	0.0091 (15)	−0.0009 (15)
C4	0.067 (2)	0.0425 (19)	0.0391 (17)	−0.0022 (17)	0.0151 (17)	−0.0064 (15)
C5	0.054 (2)	0.0388 (17)	0.0325 (15)	0.0027 (14)	0.0149 (14)	0.0075 (13)

### catena-poly[[aquachloridomanganese(II)]-di-µ-chlorido-[aquachloridomanganese(II)]-bis(µ-pyridine N-oxide)] (I) Geometric parameters (Å, º)

**Table:**

Mn1—Cl1	2.479 (2)	C1—C2	1.376 (5)
Mn1—O1	2.177 (3)	O2—H2A	0.833 (19)
Mn1—O1^i^	2.182 (2)	O2—H2B	0.819 (19)
Mn1—Cl2^ii^	2.5324 (19)	C2—H2	0.9300
Mn1—Cl2	2.5240 (19)	C2—C3	1.353 (5)
Mn1—O2	2.250 (3)	C3—H3	0.9300
O1—N1	1.341 (3)	C3—C4	1.364 (5)
N1—C1	1.331 (4)	C4—H4	0.9300
N1—C5	1.339 (4)	C4—C5	1.377 (4)
C1—H1	0.9300	C5—H5	0.9300
			
Cl1—Mn1—Cl2	93.43 (6)	C5—N1—O1	118.0 (2)
Cl1—Mn1—Cl2^ii^	92.57 (6)	N1—C1—H1	120.4
O1^i^—Mn1—Cl1	95.12 (8)	N1—C1—C2	119.1 (3)
O1—Mn1—Cl1	93.65 (7)	C2—C1—H1	120.4
O1—Mn1—O1^i^	72.02 (10)	Mn1—Cl2—Mn1^ii^	84.42 (7)
O1—Mn1—Cl2^ii^	165.77 (6)	Mn1—O2—H2A	108 (3)
O1^i^—Mn1—Cl2^ii^	94.69 (9)	Mn1—O2—H2B	116 (3)
O1^i^—Mn1—Cl2	166.32 (5)	H2A—O2—H2B	110 (4)
O1—Mn1—Cl2	96.81 (8)	C1—C2—H2	119.7
O1—Mn1—O2	86.96 (9)	C3—C2—C1	120.5 (3)
O1^i^—Mn1—O2	87.46 (10)	C3—C2—H2	119.7
Cl2—Mn1—Cl2^ii^	95.58 (7)	C2—C3—H3	120.4
O2—Mn1—Cl1	177.42 (7)	C2—C3—C4	119.1 (3)
O2—Mn1—Cl2^ii^	87.40 (8)	C4—C3—H3	120.4
O2—Mn1—Cl2	84.01 (9)	C3—C4—H4	120.0
Mn1—O1—Mn1^i^	107.98 (10)	C3—C4—C5	120.1 (3)
N1—O1—Mn1	123.78 (14)	C5—C4—H4	120.0
N1—O1—Mn1^i^	126.50 (16)	N1—C5—C4	119.1 (3)
C1—N1—O1	119.9 (2)	N1—C5—H5	120.5
C1—N1—C5	122.0 (3)	C4—C5—H5	120.5

Symmetry codes: (i) −*x*+1, −*y*+1, −*z*+1; (ii) −*x*, −*y*+1, −*z*+1.

### catena-poly[[aquachloridomanganese(II)]-di-µ-chlorido-[aquachloridomanganese(II)]-bis(µ-pyridine N-oxide)] (I) Hydrogen-bond geometry (Å, º)

**Table:**

*D*—H···*A*	*D*—H	H···*A*	*D*···*A*	*D*—H···*A*
O2—H2*A*···Cl1^ii^	0.83 (2)	2.53 (2)	3.348 (4)	168 (4)
O2—H2*B*···Cl1^iii^	0.82 (2)	2.52 (3)	3.232 (4)	147 (4)

Symmetry codes: (ii) −*x*, −*y*+1, −*z*+1; (iii) *x*, *y*−1, *z*.

### catena-Poly[[aquachloridomanganese(II)]-di-µ-chlorido-[aquachloridomanganese(II)]-bis(µ-2-methylpyridine N-oxide)] (II) Crystal data

**Table:**

[MnCl_2_(C_6_H_7_NO)(H_2_O)]	*D*_x_ = 1.811 Mg m^−^^3^
*M**_r_* = 252.98	Mo *K*α radiation, λ = 0.71075 Å
Orthorhombic, *P*2_1_2_1_2_1_	Cell parameters from 2686 reflections
*a* = 6.862 (2) Å	θ = 2.7–27.5°
*b* = 7.491 (2) Å	µ = 1.96 mm^−^^1^
*c* = 18.047 (5) Å	*T* = 173 K
*V* = 927.7 (4) Å^3^	Prism, colorless
*Z* = 4	0.2 × 0.2 × 0.1 mm
*F*(000) = 508	

### catena-Poly[[aquachloridomanganese(II)]-di-µ-chlorido-[aquachloridomanganese(II)]-bis(µ-2-methylpyridine N-oxide)] (II) Data collection

**Table:**

Rigaku XtalLab mini CCD diffractometer	1800 reflections with *I* > 2σ(*I*)
ω scans	*R*_int_ = 0.051
Absorption correction: multi-scan (REQAB; Rigaku, 1998)	θ_max_ = 27.5°, θ_min_ = 2.3°
*T*_min_ = 0.563, *T*_max_ = 0.737	*h* = −8→8
8438 measured reflections	*k* = −9→9
2109 independent reflections	*l* = −23→23

### catena-Poly[[aquachloridomanganese(II)]-di-µ-chlorido-[aquachloridomanganese(II)]-bis(µ-2-methylpyridine N-oxide)] (II) Refinement

**Table:**

Refinement on *F*^2^	Hydrogen site location: mixed
Least-squares matrix: full	H-atom parameters constrained
*R*[*F*^2^ > 2σ(*F*^2^)] = 0.051	*w* = 1/[σ^2^(*F*_o_^2^) + (0.004*P*)^2^ + 2.3909*P*] where *P* = (*F*_o_^2^ + 2*F*_c_^2^)/3
*wR*(*F*^2^) = 0.100	(Δ/σ)_max_ < 0.001
*S* = 1.12	Δρ_max_ = 0.95 e Å^−^^3^
2109 reflections	Δρ_min_ = −0.73 e Å^−^^3^
112 parameters	Absolute structure: Refined as an inversion twin.
0 restraints	Absolute structure parameter: 0.44 (8)
Primary atom site location: dual	

### catena-Poly[[aquachloridomanganese(II)]-di-µ-chlorido-[aquachloridomanganese(II)]-bis(µ-2-methylpyridine N-oxide)] (II) Special details

**Table:**

Geometry. All esds (except the esd in the dihedral angle between two l.s. planes) are estimated using the full covariance matrix. The cell esds are taken into account individually in the estimation of esds in distances, angles and torsion angles; correlations between esds in cell parameters are only used when they are defined by crystal symmetry. An approximate (isotropic) treatment of cell esds is used for estimating esds involving l.s. planes.
Refinement. Refined as a 2-component inversion twin.

### catena-Poly[[aquachloridomanganese(II)]-di-µ-chlorido-[aquachloridomanganese(II)]-bis(µ-2-methylpyridine N-oxide)] (II) Fractional atomic coordinates and isotropic or equivalent isotropic displacement parameters (Å^2^)

**Table:**

	*x*	*y*	*z*	*U*_iso_*/*U*_eq_	
Mn1	0.5059 (2)	0.73290 (15)	0.50137 (7)	0.0197 (2)	
Cl1	0.2537 (3)	0.6800 (2)	0.40260 (8)	0.0226 (3)	
O1	0.7561 (8)	0.7085 (6)	0.42855 (19)	0.0179 (9)	
N1	0.7503 (11)	0.7249 (7)	0.3544 (2)	0.0199 (11)	
C1	0.7553 (14)	0.5782 (9)	0.3112 (3)	0.0276 (15)	
Cl2	0.5309 (3)	0.4110 (3)	0.53742 (11)	0.0325 (5)	
O2	0.4833 (8)	1.0223 (7)	0.4655 (3)	0.0265 (12)	
H2A	0.546895	1.093811	0.497004	0.040*	
H2B	0.358601	1.056880	0.464095	0.040*	
C2	0.7532 (15)	0.6041 (10)	0.2345 (3)	0.0321 (16)	
H2	0.758660	0.503891	0.202342	0.038*	
C3	0.7436 (16)	0.7719 (11)	0.2056 (4)	0.0370 (18)	
H3	0.740766	0.787849	0.153377	0.044*	
C4	0.7378 (16)	0.9180 (10)	0.2515 (4)	0.0371 (19)	
H4	0.731159	1.035141	0.231495	0.044*	
C5	0.7418 (15)	0.8925 (9)	0.3261 (3)	0.0283 (16)	
H5	0.738625	0.992359	0.358479	0.034*	
C6	0.7629 (17)	0.4059 (10)	0.3484 (4)	0.040 (2)	
H6A	0.757534	0.310158	0.311520	0.048*	
H6B	0.651845	0.395059	0.382265	0.048*	
H6C	0.884583	0.396660	0.376629	0.048*	

### catena-Poly[[aquachloridomanganese(II)]-di-µ-chlorido-[aquachloridomanganese(II)]-bis(µ-2-methylpyridine N-oxide)] (II) Atomic displacement parameters (Å^2^)

**Table:**

	*U*^11^	*U*^22^	*U*^33^	*U*^12^	*U*^13^	*U*^23^
Mn1	0.0171 (4)	0.0266 (5)	0.0154 (4)	−0.0028 (5)	0.0017 (4)	−0.0008 (4)
Cl1	0.0212 (7)	0.0300 (9)	0.0167 (6)	0.0003 (10)	0.0004 (8)	−0.0069 (6)
O1	0.020 (2)	0.025 (3)	0.0089 (16)	−0.005 (3)	−0.002 (2)	−0.0013 (16)
N1	0.019 (2)	0.029 (3)	0.012 (2)	0.002 (3)	0.002 (3)	−0.001 (2)
C1	0.028 (4)	0.028 (4)	0.026 (3)	0.003 (5)	−0.001 (4)	−0.005 (3)
Cl2	0.0377 (12)	0.0234 (10)	0.0365 (11)	−0.0021 (10)	0.0037 (9)	0.0050 (8)
O2	0.015 (3)	0.048 (3)	0.016 (2)	−0.009 (3)	0.002 (2)	−0.003 (2)
C2	0.036 (4)	0.046 (5)	0.015 (3)	0.007 (6)	0.002 (4)	−0.004 (3)
C3	0.048 (5)	0.046 (5)	0.017 (3)	0.004 (6)	0.001 (4)	0.001 (3)
C4	0.060 (5)	0.027 (4)	0.024 (4)	0.002 (6)	0.003 (5)	0.005 (3)
C5	0.042 (4)	0.026 (4)	0.017 (3)	0.007 (5)	0.004 (4)	−0.002 (3)
C6	0.061 (6)	0.029 (4)	0.029 (4)	0.007 (6)	0.001 (5)	0.000 (3)

### catena-Poly[[aquachloridomanganese(II)]-di-µ-chlorido-[aquachloridomanganese(II)]-bis(µ-2-methylpyridine N-oxide)] (II) Geometric parameters (Å, º)

**Table:**

Mn1—Cl1^i^	2.514 (3)	O2—H2B	0.8947
Mn1—Cl1	2.516 (4)	C2—H2	0.9500
Mn1—O1^ii^	2.174 (5)	C2—C3	1.363 (10)
Mn1—O1	2.171 (6)	C3—H3	0.9500
Mn1—Cl2	2.503 (4)	C3—C4	1.374 (10)
Mn1—O2	2.268 (6)	C4—H4	0.9500
O1—N1	1.345 (6)	C4—C5	1.360 (9)
N1—C1	1.348 (8)	C5—H5	0.9500
N1—C5	1.357 (8)	C6—H6A	0.9800
C1—C2	1.397 (9)	C6—H6B	0.9800
C1—C6	1.456 (10)	C6—H6C	0.9800
O2—H2A	0.8951		
			
Cl1^i^—Mn1—Cl1	174.01 (5)	N1—C1—C6	117.1 (6)
O1^ii^—Mn1—Cl1	84.38 (18)	C2—C1—C6	125.5 (7)
O1—Mn1—Cl1^i^	84.49 (18)	Mn1—O2—H2A	110.9
O1^ii^—Mn1—Cl1^i^	94.57 (18)	Mn1—O2—H2B	110.5
O1—Mn1—Cl1	95.84 (18)	H2A—O2—H2B	108.1
O1—Mn1—O1^ii^	173.11 (6)	C1—C2—H2	119.7
O1—Mn1—Cl2	91.26 (14)	C3—C2—C1	120.5 (7)
O1^ii^—Mn1—Cl2	95.58 (14)	C3—C2—H2	119.7
O1^ii^—Mn1—O2	85.39 (19)	C2—C3—H3	119.8
O1—Mn1—O2	87.78 (19)	C2—C3—C4	120.3 (6)
Cl2—Mn1—Cl1^i^	91.40 (9)	C4—C3—H3	119.8
Cl2—Mn1—Cl1	94.57 (9)	C3—C4—H4	120.5
O2—Mn1—Cl1	84.34 (16)	C5—C4—C3	119.0 (7)
O2—Mn1—Cl1^i^	89.71 (16)	C5—C4—H4	120.5
O2—Mn1—Cl2	178.46 (15)	N1—C5—C4	120.3 (6)
Mn1^ii^—Cl1—Mn1	86.32 (13)	N1—C5—H5	119.9
Mn1—O1—Mn1^i^	104.73 (19)	C4—C5—H5	119.9
N1—O1—Mn1^i^	125.7 (5)	C1—C6—H6A	109.5
N1—O1—Mn1	124.9 (5)	C1—C6—H6B	109.5
O1—N1—C1	120.1 (5)	C1—C6—H6C	109.5
O1—N1—C5	117.4 (5)	H6A—C6—H6B	109.5
C1—N1—C5	122.5 (5)	H6A—C6—H6C	109.5
N1—C1—C2	117.4 (7)	H6B—C6—H6C	109.5
			
Mn1—O1—N1—C1	−102.4 (8)	C1—N1—C5—C4	0.0 (15)
Mn1^i^—O1—N1—C1	105.6 (8)	C1—C2—C3—C4	0.7 (17)
Mn1—O1—N1—C5	78.4 (9)	C2—C3—C4—C5	0.0 (17)
Mn1^i^—O1—N1—C5	−73.7 (9)	C3—C4—C5—N1	−0.3 (17)
O1—N1—C1—C2	−178.6 (8)	C5—N1—C1—C2	0.6 (14)
O1—N1—C1—C6	1.2 (13)	C5—N1—C1—C6	−179.6 (10)
O1—N1—C5—C4	179.3 (9)	C6—C1—C2—C3	179.2 (11)
N1—C1—C2—C3	−1.0 (16)		

Symmetry codes: (i) *x*+1/2, −*y*+3/2, −*z*+1; (ii) *x*−1/2, −*y*+3/2, −*z*+1.

### catena-Poly[[aquachloridomanganese(II)]-di-µ-chlorido-[aquachloridomanganese(II)]-bis(µ-2-methylpyridine N-oxide)] (II) Hydrogen-bond geometry (Å, º)

**Table:**

*D*—H···*A*	*D*—H	H···*A*	*D*···*A*	*D*—H···*A*
O2—H2*A*···Cl2^iii^	0.90	2.49	3.205 (7)	137
O2—H2*B*···Cl2^ii^	0.89	2.26	3.145 (7)	169

Symmetry codes: (ii) *x*−1/2, −*y*+3/2, −*z*+1; (iii) *x*, *y*+1, *z*.

### Bis(µ-3-methylpyridine N-oxide)bis[diaquadichloridomanganese(II)] (III) Crystal data

**Table:**

[Mn_2_Cl_4_(C_6_H_7_NO)_2_(H_2_O)_4_]	*Z* = 1
*M**_r_* = 541.99	*F*(000) = 274
Triclinic, *P*1	*D*_x_ = 1.629 Mg m^−^^3^
*a* = 7.902 (7) Å	Mo *K*α radiation, λ = 0.71073 Å
*b* = 8.026 (7) Å	Cell parameters from 5886 reflections
*c* = 9.893 (8) Å	θ = 2.8–27.5°
α = 98.033 (1)°	µ = 1.65 mm^−^^1^
β = 99.272 (7)°	*T* = 173 K
γ = 113.634 (11)°	Prism, clear brown
*V* = 552.6 (8) Å^3^	0.85 × 0.5 × 0.28 mm

### Bis(µ-3-methylpyridine N-oxide)bis[diaquadichloridomanganese(II)] (III) Data collection

**Table:**

Rigaku XtalLab mini CCD diffractometer	2375 reflections with *I* > 2σ(*I*)
ω scans	*R*_int_ = 0.072
Absorption correction: multi-scan (REQAB; Rigaku, 1998)	θ_max_ = 27.6°, θ_min_ = 2.8°
*T*_min_ = 0.482, *T*_max_ = 0.630	*h* = −10→10
5837 measured reflections	*k* = −10→10
2553 independent reflections	*l* = −12→12

### Bis(µ-3-methylpyridine N-oxide)bis[diaquadichloridomanganese(II)] (III) Refinement

**Table:**

Refinement on *F*^2^	Primary atom site location: dual
Least-squares matrix: full	Hydrogen site location: mixed
*R*[*F*^2^ > 2σ(*F*^2^)] = 0.031	H atoms treated by a mixture of independent and constrained refinement
*wR*(*F*^2^) = 0.087	*w* = 1/[σ^2^(*F*_o_^2^) + (0.031*P*)^2^ + 0.023*P*] where *P* = (*F*_o_^2^ + 2*F*_c_^2^)/3
*S* = 1.07	(Δ/σ)_max_ < 0.001
2553 reflections	Δρ_max_ = 0.56 e Å^−^^3^
135 parameters	Δρ_min_ = −0.41 e Å^−^^3^
4 restraints	

### Bis(µ-3-methylpyridine N-oxide)bis[diaquadichloridomanganese(II)] (III) Special details

**Table:**

Geometry. All esds (except the esd in the dihedral angle between two l.s. planes) are estimated using the full covariance matrix. The cell esds are taken into account individually in the estimation of esds in distances, angles and torsion angles; correlations between esds in cell parameters are only used when they are defined by crystal symmetry. An approximate (isotropic) treatment of cell esds is used for estimating esds involving l.s. planes.

### Bis(µ-3-methylpyridine N-oxide)bis[diaquadichloridomanganese(II)] (III) Fractional atomic coordinates and isotropic or equivalent isotropic displacement parameters (Å^2^)

**Table:**

	*x*	*y*	*z*	*U*_iso_*/*U*_eq_	
Mn1	0.27031 (3)	0.40104 (3)	0.53553 (2)	0.02936 (11)	
Cl1	0.14922 (6)	0.48607 (7)	0.73553 (4)	0.04342 (14)	
Cl2	0.32299 (6)	0.13672 (6)	0.60668 (6)	0.04544 (15)	
O1	0.56562 (15)	0.60090 (16)	0.62683 (12)	0.0345 (3)	
O2	0.2605 (2)	0.6467 (2)	0.45620 (16)	0.0443 (3)	
H2A	0.355 (3)	0.710 (3)	0.436 (2)	0.055 (7)*	
H2B	0.167 (3)	0.639 (3)	0.404 (2)	0.062 (8)*	
O3	0.00694 (17)	0.23434 (18)	0.37696 (14)	0.0395 (3)	
H3A	−0.069 (3)	0.128 (2)	0.390 (2)	0.062 (7)*	
H3B	−0.054 (4)	0.288 (4)	0.352 (3)	0.081 (10)*	
N1	0.62886 (17)	0.71302 (18)	0.75570 (13)	0.0294 (3)	
C1	0.6561 (2)	0.8903 (2)	0.77007 (17)	0.0342 (4)	
H1	0.631326	0.934009	0.690425	0.041*	
C2	0.7204 (3)	1.0102 (3)	0.90084 (19)	0.0415 (4)	
C3	0.7538 (3)	0.9400 (3)	1.0169 (2)	0.0568 (6)	
H3	0.795802	1.016517	1.106619	0.068*	
C4	0.7250 (4)	0.7571 (4)	0.9997 (2)	0.0611 (6)	
H4	0.748138	0.710090	1.077885	0.073*	
C5	0.6620 (3)	0.6431 (3)	0.8670 (2)	0.0472 (5)	
H5	0.642729	0.519251	0.854911	0.057*	
C6	0.7494 (4)	1.2087 (3)	0.9131 (3)	0.0701 (7)	
H6A	0.873126	1.282752	0.900126	0.105*	
H6B	0.739622	1.256760	1.004510	0.105*	
H6C	0.653880	1.213706	0.842403	0.105*	

### Bis(µ-3-methylpyridine N-oxide)bis[diaquadichloridomanganese(II)] (III) Atomic displacement parameters (Å^2^)

**Table:**

	*U*^11^	*U*^22^	*U*^33^	*U*^12^	*U*^13^	*U*^23^
Mn1	0.02821 (16)	0.02780 (17)	0.03004 (17)	0.00959 (12)	0.00843 (11)	0.00651 (12)
Cl1	0.0471 (3)	0.0617 (3)	0.0309 (2)	0.0299 (2)	0.01431 (18)	0.0142 (2)
Cl2	0.0436 (3)	0.0349 (3)	0.0596 (3)	0.0137 (2)	0.0151 (2)	0.0236 (2)
O1	0.0326 (6)	0.0310 (6)	0.0313 (6)	0.0074 (5)	0.0091 (5)	−0.0010 (5)
O2	0.0389 (7)	0.0414 (7)	0.0584 (9)	0.0185 (6)	0.0137 (6)	0.0231 (7)
O3	0.0339 (6)	0.0322 (7)	0.0438 (7)	0.0079 (5)	0.0039 (5)	0.0080 (6)
N1	0.0295 (6)	0.0293 (7)	0.0271 (6)	0.0107 (5)	0.0061 (5)	0.0062 (5)
C1	0.0443 (9)	0.0307 (8)	0.0273 (7)	0.0159 (7)	0.0082 (6)	0.0067 (6)
C2	0.0484 (10)	0.0363 (9)	0.0336 (8)	0.0138 (8)	0.0112 (7)	0.0016 (7)
C3	0.0674 (13)	0.0598 (13)	0.0262 (9)	0.0158 (11)	0.0049 (9)	0.0014 (9)
C4	0.0766 (14)	0.0687 (15)	0.0355 (10)	0.0289 (12)	0.0028 (10)	0.0246 (10)
C5	0.0591 (11)	0.0424 (10)	0.0425 (10)	0.0236 (9)	0.0064 (9)	0.0186 (9)
C6	0.105 (2)	0.0393 (11)	0.0571 (14)	0.0257 (13)	0.0237 (14)	−0.0048 (10)

### Bis(µ-3-methylpyridine N-oxide)bis[diaquadichloridomanganese(II)] (III) Geometric parameters (Å, º)

**Table:**

Mn1—Cl1	2.4602 (15)	C1—H1	0.9300
Mn1—Cl2	2.4900 (19)	C1—C2	1.381 (2)
Mn1—O1^i^	2.2228 (17)	C2—C3	1.381 (3)
Mn1—O1	2.1792 (18)	C2—C6	1.500 (3)
Mn1—O2	2.246 (2)	C3—H3	0.9300
Mn1—O3	2.1704 (17)	C3—C4	1.373 (4)
O1—N1	1.3411 (19)	C4—H4	0.9300
O2—H2A	0.791 (15)	C4—C5	1.377 (3)
O2—H2B	0.802 (16)	C5—H5	0.9300
O3—H3A	0.863 (16)	C6—H6A	0.9600
O3—H3B	0.795 (17)	C6—H6B	0.9600
N1—C1	1.334 (2)	C6—H6C	0.9600
N1—C5	1.340 (2)		
			
Cl1—Mn1—Cl2	98.31 (4)	C1—N1—O1	119.45 (13)
O1—Mn1—Cl1	95.44 (6)	C1—N1—C5	121.69 (16)
O1^i^—Mn1—Cl1	165.45 (4)	C5—N1—O1	118.86 (16)
O1^i^—Mn1—Cl2	89.66 (5)	N1—C1—H1	119.3
O1—Mn1—Cl2	93.11 (7)	N1—C1—C2	121.31 (16)
O1—Mn1—O1^i^	71.87 (7)	C2—C1—H1	119.3
O1^i^—Mn1—O2	82.07 (6)	C1—C2—C3	117.8 (2)
O1—Mn1—O2	81.64 (7)	C1—C2—C6	119.87 (19)
O2—Mn1—Cl1	89.20 (6)	C3—C2—C6	122.4 (2)
O2—Mn1—Cl2	171.24 (4)	C2—C3—H3	120.0
O3—Mn1—Cl1	101.10 (7)	C4—C3—C2	119.99 (19)
O3—Mn1—Cl2	97.13 (6)	C4—C3—H3	120.0
O3—Mn1—O1^i^	89.88 (8)	C3—C4—H4	119.9
O3—Mn1—O1	159.02 (5)	C3—C4—C5	120.16 (18)
O3—Mn1—O2	85.76 (7)	C5—C4—H4	119.9
Mn1—O1—Mn1^i^	108.13 (7)	N1—C5—C4	119.10 (19)
N1—O1—Mn1	124.29 (9)	N1—C5—H5	120.5
N1—O1—Mn1^i^	127.41 (10)	C4—C5—H5	120.5
Mn1—O2—H2A	114.4 (17)	C2—C6—H6A	109.5
Mn1—O2—H2B	121.9 (18)	C2—C6—H6B	109.5
H2A—O2—H2B	112 (2)	C2—C6—H6C	109.5
Mn1—O3—H3A	118.3 (16)	H6A—C6—H6B	109.5
Mn1—O3—H3B	116 (2)	H6A—C6—H6C	109.5
H3A—O3—H3B	109 (3)	H6B—C6—H6C	109.5

Symmetry code: (i) −*x*+1, −*y*+1, −*z*+1.

### Bis(µ-3-methylpyridine N-oxide)bis[diaquadichloridomanganese(II)] (III) Hydrogen-bond geometry (Å, º)

**Table:**

*D*—H···*A*	*D*—H	H···*A*	*D*···*A*	*D*—H···*A*
O2—H2*B*···Cl1^ii^	0.80 (2)	2.38 (2)	3.147 (3)	161 (2)
O3—H3*A*···Cl2^iii^	0.86 (2)	2.28 (2)	3.120 (2)	167 (2)

Symmetry codes: (ii) −*x*, −*y*+1, −*z*+1; (iii) −*x*, −*y*, −*z*+1.
